# Biofilm Inhibition by Novel Natural Product- and Biocide-Containing Coatings Using High-Throughput Screening

**DOI:** 10.3390/ijms19051434

**Published:** 2018-05-10

**Authors:** Maria Salta, Simon P. Dennington, Julian A. Wharton

**Affiliations:** 1 Faculty of Sciences, School of Biological Sciences, University of Portsmouth, Portsmouth PO1 2DY, UK; 2 Faculty of Engineering and the Environment, National Centre for Advanced Tribology at Southampton (nCATS), University of Southampton, Highfield, Southampton SO17 1BJ, UK; S.P.Dennington@soton.ac.uk (S.P.D.); j.a.wharton@soton.ac.uk (J.A.W.)

**Keywords:** biofilms, antifouling coatings, biocides, bioassay, natural products

## Abstract

The use of natural products (NPs) as possible alternative biocidal compounds for use in antifouling coatings has been the focus of research over the past decades. Despite the importance of this field, the efficacy of a given NP against biofilm (mainly bacteria and diatoms) formation is tested with the NP being in solution, while almost no studies test the effect of an NP once incorporated into a coating system. The development of a novel bioassay to assess the activity of NP-containing and biocide-containing coatings against marine biofilm formation has been achieved using a high-throughput microplate reader and highly sensitive confocal laser scanning microscopy (CLSM), as well as nucleic acid staining. Juglone, an isolated NP that has previously shown efficacy against bacterial attachment, was incorporated into a simple coating matrix. Biofilm formation over 48 h was assessed and compared against coatings containing the NP and the commonly used booster biocide, cuprous oxide. Leaching of the NP from the coating was quantified at two time points, 24 h and 48 h, showing evidence of both juglone and cuprous oxide being released. Results from the microplate reader showed that the NP coatings exhibited antifouling efficacy, significantly inhibiting biofilm formation when compared to the control coatings, while NP coatings and the cuprous oxide coatings performed equally well. CLSM results and COMSTAT analysis on biofilm 3D morphology showed comparable results when the NP coatings were tested against the controls, with higher biofilm biovolume and maximum thickness being found on the controls. This new method proved to be repeatable and insightful and we believe it is applicable in antifouling and other numerous applications where interactions between biofilm formation and surfaces is of interest.

## 1. Introduction

Biofouling, the accumulation of marine organisms on a surface, severely affects manmade structures such as ship hulls, energy systems, environmental sensors, aquaculture settings, etc. A number of technological advances focusing mainly on coating these structures with antifouling agents, have been utilised over the last few decades, with the use of biocides being the most successful. However, the biocide with the best antifouling (AF) efficacy against a wide range of target species, known as tributyltin (TBT), has been found to be detrimental to the marine environment [1,2]. Since TBT’s complete ban in 2008, there has been increased effort to discover new compounds that would perform equally well but with no environmental impact. Screening of natural products (NPs) as potential AF agents is an ongoing interest within the scientific community and industry [3,4,5,6]. An increasingly important target within the AF community is the inhibition of biofilm growth on manmade surfaces. Biofilms, which are composed mainly of bacteria and diatoms embedded within extracellular polymeric substances (EPS), are the first type of biofouling to colonise a surface, while a growing body of studies supports the position that biofilms attract larger organisms to settle upon them [7]. Biofilms are tremendously costly to society, as they adversely affect numerous industries ranging from petrochemicals to health care. Currently there are several high-throughput methods that allow the simultaneous evaluation/screening of new agents for their AF efficacy at a range of concentrations and against various marine target species [8,9]. These laboratory methods are necessary to determine whether a compound may be considered for further evaluation. However, there is a lack of studies for determining their effect once incorporated into a coating system. Stafslien et al. [10,11] have developed a rapid and high-throughput laboratory-based coating testing system, which, however, requires highly specified equipment that is uniquely available to their laboratory.

The first objective of the current work was to develop a bioassay for the rapid in situ assessment of biofilm formation (model marine species: *Cobetia marina*) on coated surfaces using universally available equipment. This was achieved by utilising 24-well plates combined with nucleic acid staining, high-throughput plate reader technology, and confocal laser scanning microscopy (CLSM). Microplate readers have the capacity to rapidly screen bacterial responses (e.g., via spectrophometric and fluorescence measurements) under different conditions and have been routinely used for the discovery of novel antimicrobials. Furthermore, CLSM allows three-dimensional (3D) visualisation of the biofilm, providing new insights into biofilm development. A few recent studies have reported the use of plate readers (microtiter plate assays) in conjunction with biological stains to measure biofilm inhibition of adhesion and/or growth within an antifouling context [12,13,14,15,16], although these studies were not designed to perform tests on antibiofilm coatings. The second objective of the current work was to use this novel bioassay for the evaluation and screening of a terrestrial NP juglone (5-hydroxy-1,4-naphthalenedione), derived from walnut trees, for potential use as a natural AF agent within a coating system. This investigation explored the proof of concept for a rapid assessment method (48 h in this case) that has the potential to be used for a broad range of antibiofilm studies, where the investigation of bacterial/surface interactions is of primary importance (i.e., antifouling industry, medical, and dental).

## 2. Results and Discussion

### 2.1. Determination of Juglone Leaching

Juglone was first dissolved in artificial seawater (ASW) in order to establish the wavelength at which the compound has maximum absorption via UV-Vis, which was found to be at 520 nm. A calibration curve was generated at this wavelength. To establish the extent of juglone leaching from the two juglone-containing coatings, tests were performed over 48 h. This was done in order to determine whether juglone actually leached from the coated surfaces, and if so, to quantify the concentrations within the 24-well fluid volumes.

During the leaching tests, two samples were taken at 24 h and 48 h. Overall, juglone leaching was detected from both coating formulations at concentrations ranging from 1.70 ppm to 1.99 ppm after 24 h, and 1.96 ppm to 2.35 ppm after 48 h, for the coating containing juglone (JUG)+poly(methyl methacrylate) (PMMA)+Rosin (ROS) and the coating JUG+PMMA, respectively (see Table 1). Overall, most of the juglone had leached out at 24 h for both coatings.

PMMA is a common acrylic polymer material also used in orthopaedic surgery as part of bone cement. Antibiotics are often incorporated into these cements, where subsequent elution produces high local concentrations of antibiotics while simultaneously minimising systemic toxicity [17,18,19]. A major characteristic of currently available antibiotic cements is that an initial burst of antibiotic elution occurs within 24 h to 48 h, with poor subsequent sustained release [20,21,22,23]. Similarly, in the current study, a burst elution when NPs are incorporated into PMMA cannot be excluded. The 24–48 h leaching window observed for antibiotics would be ideal for AF bioassays, as biofilm growth can easily be supported within that time scale. Biofilm-mediated release rates of biocides have been previously proposed, as the natural bacterial extrapolymeric matrix has shown to bind metals such as copper [24] and potentially affect the diffusion rate of the compound and consequently control its release rate [25].

### 2.2. MIC, EC_50_, and MBC for Juglone in Solution

**Figure 1:**
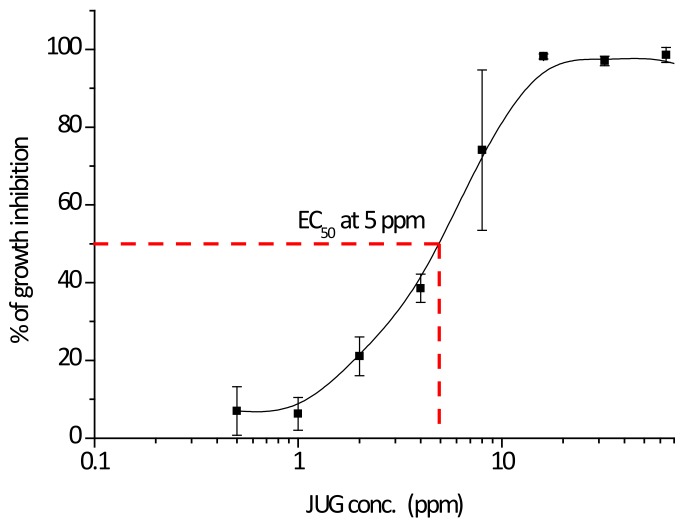
Inhibition of growth for *C. marina* (Absorbance, OD_595_) in the presence of juglone at concentrations 64, 32, 16, 8, 4, 2, 1, and 0.5 ppm (*x*-axis). Error bars ±STDEV.

The growth-inhibiting activity of juglone was examined against planktonic growth of the model species *Cobetia marina* (ATCC 25374). *C. marina* has previously been used as model species for marine biofilm related studies [7,13,15,26,27]. It was found to have strong inhibition against *C. marina* at 16 ppm, while the EC_50_ was found to be at 5 ppm, Figure 1. This is in good agreement with previous work where bacterial attachment for the same species was assessed [15]. The minimum bactericidal concentration (MBC) for *C. marina* was found to be at 16 ppm. The measured concentration of released juglone, as shown in the previous section, falls within the same range as the concentration for the EC_50_ for this species.

### 2.3. Assessing Biofilm Growth on Antibiofilm Coatings Using the Microplate Reader

**Figure 2:**
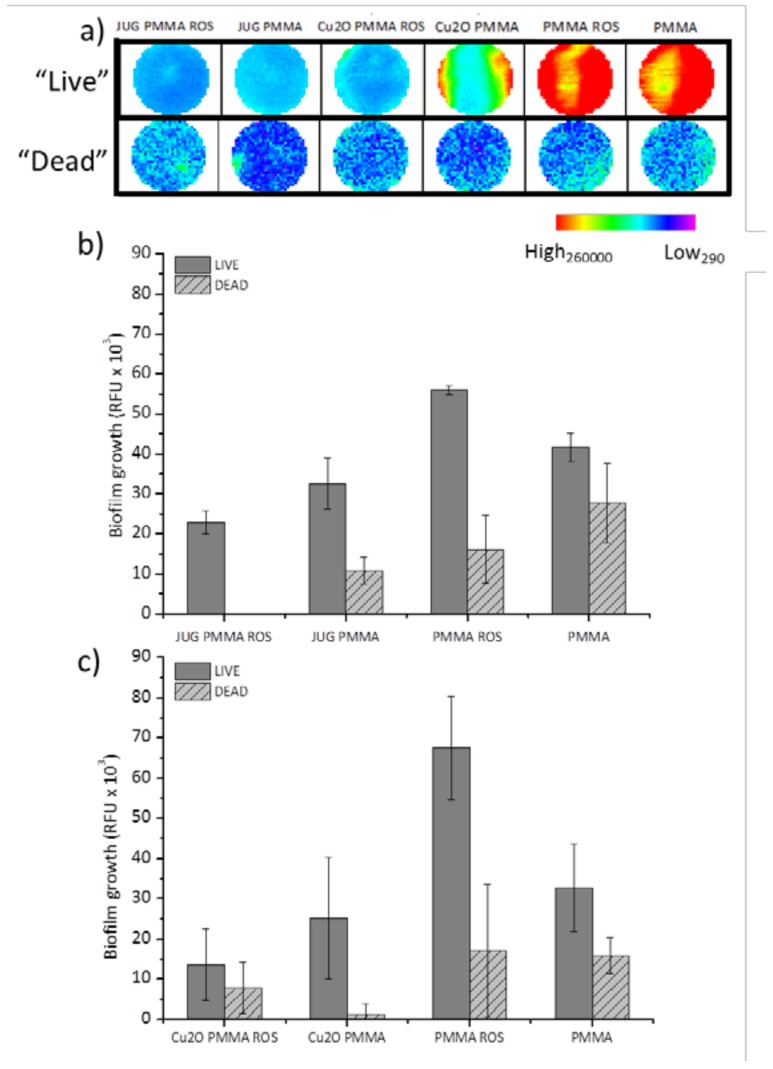
Biofilm growth/adhesion after 48 h using LIVE/DEAD (Syto9/PI) nucleic acid staining on coated coupons: (**a**) representative well-scanning images (213 × 213 μm scanning matrix) of the in situ fluorescence measurements; (**b**) average fluorescence signals from NP coatings and controls and (**c**) average fluorescence signals from biocidal coatings and controls. JUG = juglone, PMMA = poly(methyl methacrylate), ROS = rosin. RFU: Relative Fluorescence Units, ±SE.

The in situ evaluation of biofilm growth on coated surfaces was achieved through the use of a plate reader utilising nucleic acid staining. In Figure 2, the effect of each coating, i.e., 2 NP, 2 biocidal, and 2 controls, on biofilm attachment and growth is shown (detailed coating formulations can be seen in Materials and Methods, Overall, from the plate reader coating scans (live cells stained with Syto9) (Figure 2a), a clear difference in biofilm growth is apparent for juglone- and Cu_2_O-containing coatings when compared to their controls, PMMA and PMMA + Rosin which acquired significantly higher growth (*p* < 0.004). For the “dead” (propidium iodide stained cells) wavelength measurements (“dead” = cells with compromised membranes), there was no significant difference between coatings and there was an overall low number of compromised cells for the duration of the experiment.

Specifically, coatings with the binder system PMMA + Rosin showed significantly higher biofilm formation when compared to the NP coating (juglones) and the biocidal coating (Cu_2_O) with *p* < 0.042 and *p* < 0.003, respectively (Figure 2b,c). For the coatings containing PMMA as the sole binder (i.e., rosin was not incorporated), no significant difference in biofilm formation was observed when comparing them against the ones containing juglone and Cu_2_O (i.e., the JUG PMMA vs. Cu_2_O PMMA vs. PMMA). This indicates that PMMA alone as a binder did not facilitate sufficient compound release and therefore did not perform as well as the binder with the rosin additive. The leaching tests (Table 1) have demonstrated that when PMMA was used as the sole binder, most of the juglone was found to be released within the first 24 h. On the other hand, the combination of PMMA + Rosin demonstrated a more controlled release with the juglone concentration accumulating throughout the test duration, i.e., reaching higher values at 48 h. The rosin addition may have allowed greater availability of the compound at the coated surface throughout the 48 h, potentially resulting in a better antibiofilm performance.

When the juglone coatings were compared against the Cu_2_O coatings, no significant difference in biofilm growth was found (Figure 2b,c). In terms of dead cell wavelengths, there appear to be differences between coatings, however, there are no clear trends. Therefore, it can be concluded that the juglone-containing coatings performed similarly to those containing the biocide (Cu_2_O), indicating a clear antibiofilm activity against *C. marina*.

### 2.4. Planktonic Growth

**Figure 3:**
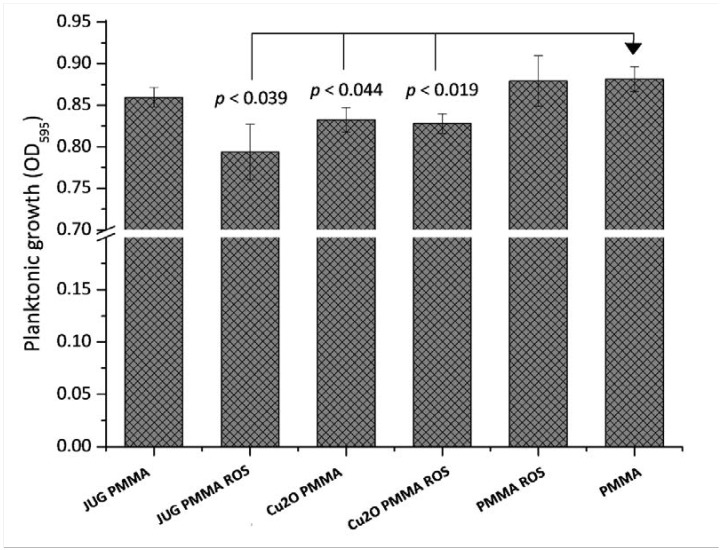
Planktonic growth of *C. marina* at the experimental end-point (48 h), taken from wells containing the coated surfaces as indicated in the *x*-axis. JUG = juglone, PMMA = poly(methyl methacrylate), ROS = rosin. Error bars ±SE.

The planktonic growth measured at the 48 h end-point within the wells containing the coated coupons can be seen in Figure 3. Interestingly, differences were also found in planktonic growth within the wells containing the different coatings. Planktonic growth was found to be significantly lower in the wells where coatings contained juglone (*p* = 0.039) and Cu_2_O (*p* = 0.044 and *p* = 0.019) when compared to the controls (PMMA ROS and PMMA), with the exception of the Juglone–PMMA combination. As shown earlier, the rosin additive performed better in releasing juglone into the solution, therefore its higher availability could account for lower bacterial growth in these wells.

### 2.5. Biofilm Morphology

**Figure 4:**
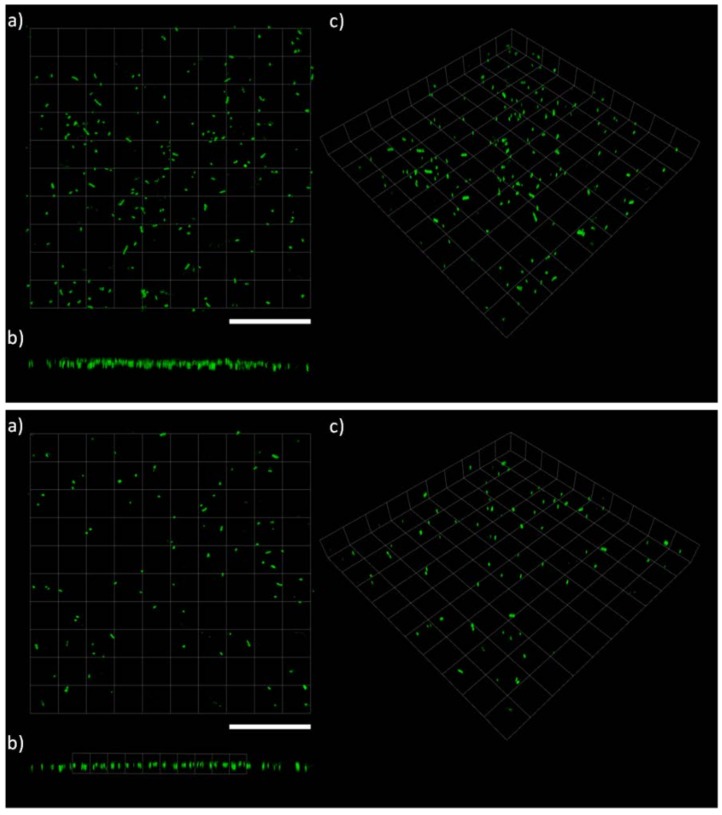
CLSM microscopy on LIVE/DEAD stained *C. marina* on the coated surfaced at the end-point (48 h), where (**Top**): JUG + PMMA + ROS, (**Bottom**): JUG + PMMA and (**a**) top view, (**b**) side view and (**c**) 3D view. Scale bars = 112 μm. JUG = juglone, PMMA = poly(methyl methacrylate), ROS = rosin.

**Figure 5:**
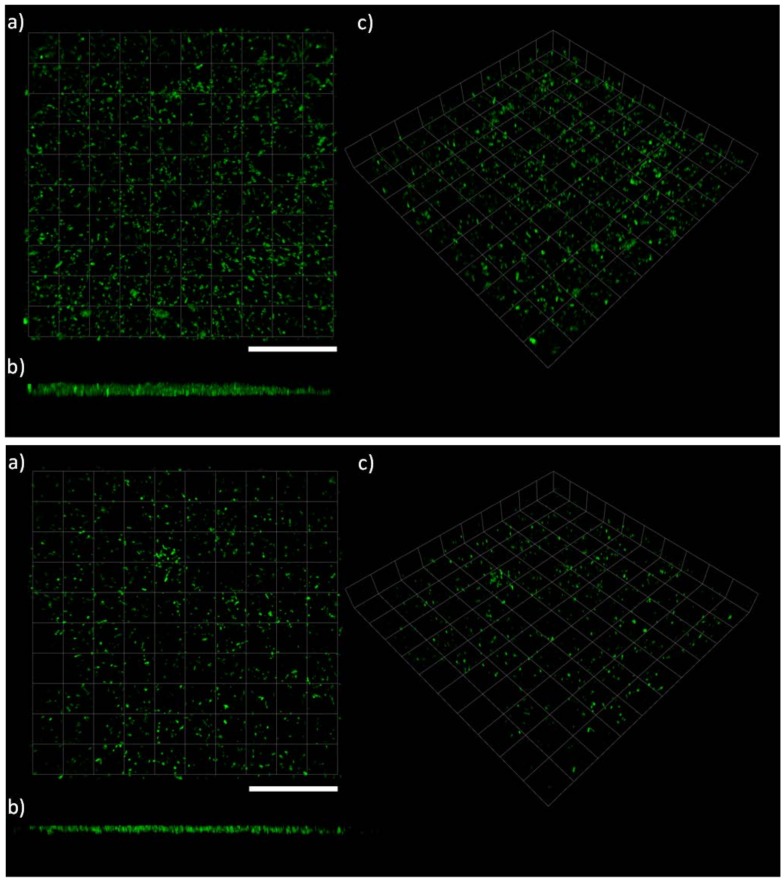
CLSM microscopy on LIVE/DEAD stained *C. marina* on the coated surfaces at the end-point (48 h), where (**Top**): Cu_2_O + PMMA + ROS, (**Bottom**): Cu_2_O + PMMA and (**a**) top view, (**b**) side view and (**c**) 3D view. Scale bars= 112 μm. PMMA = poly(methyl methacrylate), ROS = rosin.

**Figure 6:**
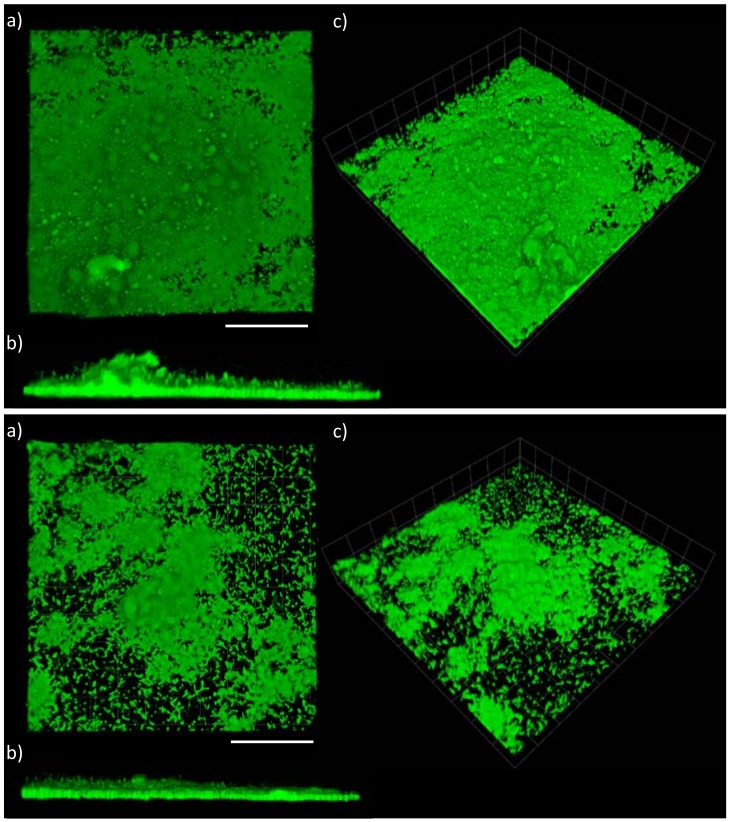
CLSM microscopy on LIVE/DEAD stained *C. marina* on the coated surfaces at the end-point (48 h), where (**Top**): PMMA + ROS, (**Bottom**): PMMA and (**a**) top view, (**b**) side view and (**c**) 3D view. Scale bars = 112 μm. PMMA = poly(methyl methacrylate), ROS = rosin.

Data analysis from the confocal laser scanning microscopy (CLSM) using COMSTAT was undertaken in order to: (*i*) confirm the plate reader results and allow direct comparisons between the two techniques and (*ii*) to utilise the high resolution of the CLSM to assess the biofilm microstructures that formed on the various coatings. Figure 4, Figure 5 and Figure 6 show examples of CLSM images obtained from 3D stacks associated with the NP, biocidal, and control coatings, respectively, and Table 2 summarises all obtained results from COMSTAT. The NP coatings (juglone) are characterised by single and/or chains of cells with an apparent lack of EPS, Figure 4. Conversely, on the control coatings (PMMA + Rosin and PMMA), clear biofilm structures can be seen, especially on the PMMA + Rosin, where a thick and relatively homogenous biofilm is formed, while on the PMMA control, evidence of clustered cells and EPS is also apparent, however to a lesser extent (when compared to PMMA + Rosin) (see Figure 6). For the biocidal coatings (Cu_2_O), no biofilm formation can be seen and the cell morphology appears to be different, with maybe a few rod-like cells and more undefined shaped particles, leading to doubts as to whether these bacterial cells are intact (Figure 5).

**Figure 7:**
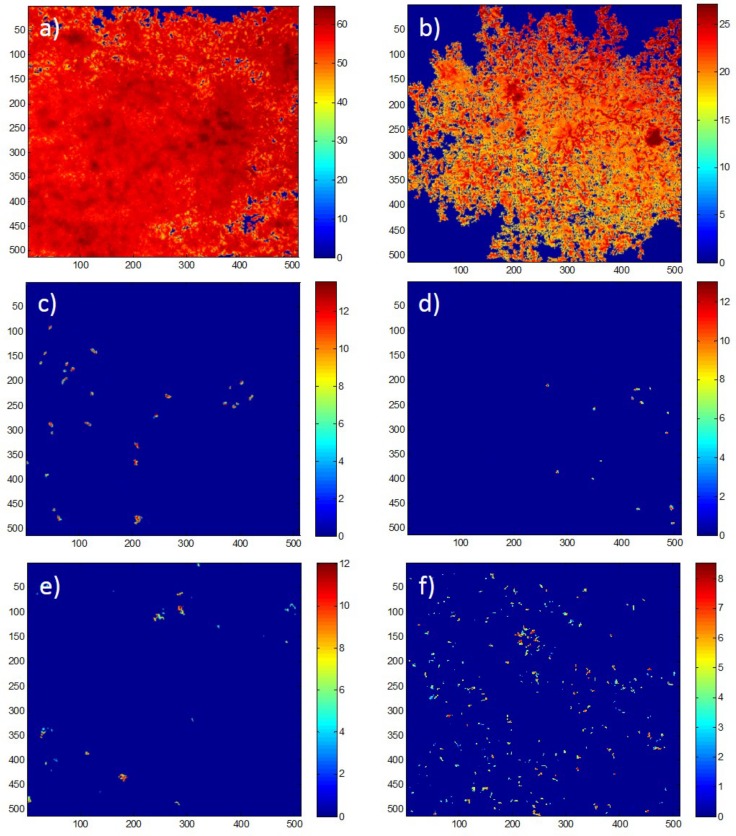
Representative COMSTAT (MATLAB) intensity map reflecting biofilm thickness (scale in μm) on (**a**) PMMA + ROS, (**b**) PMMA, (**c**) PMMA+ ROS + JUG, (**d**) PMMA + JUG, (**e**) PMMA + ROS + Cu_2_O, f) PMMA + Cu_2_O. JUG = juglone, PMMA = poly(methyl methacrylate), ROS = rosin.

Specifically, there was significantly higher biofilm thickness (Figure 7) and biovolume (*p* < 0.001 for both parameters) on the control coatings PMMA + Rosin when compared to the juglone and Cu_2_O coatings. These results are in good agreement with the data obtained from the plate reader (Table 2). The same significant effect (*p* < 0.002) of the PMMA controls on biofilm morphology was also observed for the JUG PMMA vs. Cu_2_O PMMA vs. PMMA (control), i.e., greater biofilm thickness (Figure 7) and biovolume (*p* < 0.001 and *p* < 0.002, respectively) were found on the PMMA control coatings (Table 2). This effect was not clearly evident from the plate reader data, although a trend could be observed. In the current work, some level of autofluorescence was observed when fluorescence measurements were taken prior to bacterial addition. However, this was accounted for, as blanks were part of the experimental design, allowing for the subtraction of background signals. Indeed, blanks are crucial in this type of experiment, as polymer-based coatings, for instance, can often autofluoresce. Here, we showed that this can be accounted for, although care must be taken to avoid any false readings due to lack of appropriate blanks in the experimental matrix.

As observed from the plate reader measurements, the CLSM data also confirmed the almost entire absence of dead cells. This implies a nontoxic effect of the coatings on biofilms, and therefore, only inhibition of attachment and growth. However, the almost complete lack of dead cells still remains to be fully understood. For instance, as described earlier, there were lower OD values accounting for the planktonic cells in the juglone and biocidal coating wells when compared to the controls implying cell death. In future experiments, planktonic cells could also be stained to assess their viability. The overall toxicity mechanism of naphthoquinones, such as juglone, has still not been clearly established, especially towards prokaryotes. However, the potential interference of juglone (which is a strong redox cycler with high potential to react with oxygen and its reactive species) on cell division and membrane transport [29,30] may account for the overall inhibition of biofilm growth on juglone-coated surfaces seen in the current work.

The use of CLSM was found to be informative on various additional biofilm characterisation parameters corroborating/adding to the less sensitive but high-throughput analysis by the plate reader. Overall, there was good consistency between the two techniques, underlining a good antibiofilm performance of the juglone-containing coatings. The few discrepancies between the two datasets may be attributed to the nature of the measurement methods, i.e., CLSM uses laser light which provides better controllability since emitted wavelengths can usually be optimised to limit overlapping of the coatings’ potential autofluorescence and the nucleic acid stains.

## 3. Materials and Methods

### 3.1. Test Surfaces and Coating Formulations

Circular glass coupons (thickness: 3.5 mm, diameter: 13.5 mm) were abraded using a lapping machine (Kemet 15 Diamond Flat Lapping/Polishing machine, Kemet International Ltd., Maidstone, UK) with 25 μm diamond microparticles for 20 min to produce rough surfaces. This was done to improve adhesion between the smooth glass surface and the coatings. Each coupon was permanently numbered and weighed. The main binder for these model coatings was poly(methyl methacrylate) (PMMA); however, the sole use of this hard acrylic polymer as an inert binder could potentially limit the release of the NP. Therefore, rosin was also added, since it is a common binder ingredient in paint formulations. The composition (matrix) of these paints must contain a material sufficiently soluble in seawater to maintain adequate leaching of the toxin, but not so soluble as to result in the rapid deterioration of the film. Rosin is commonly used for this purpose since its solubility appears to be within the limits required (e.g., [31]). Details on composition of the coating formulations applied are listed in Table 3.

Polymer solutions for the coatings were prepared by dissolving solid polymer (PMMA at 30 wt. %) in toluene solvent (laboratory reagent grade, low in sulphur, Fisher Scientific, CAS: 108-88-3, Hampton, NH, USA) under constant stirring. Rosin (gum rosin, Aldrich, CAS: 8050-09-7, St. Louis, MS, USA) was dissolved rapidly in toluene under mild agitation at ambient temperature, while dissolution of PMMA (M. Wt. 120,000 by GPC, Sigma-Aldrich, CAS: 1317-39-1, St. Louis, MO, USA) was slower and was facilitated by heating the mixture of polymer powder and toluene at 50 °C in a reaction flask fitted with a reflux condenser and stirring with a paddle stirrer at 60 rpm. To make the 80:20 wt./wt. PMMA:rosin polymer blend, solutions of each polymer having the same solid content (30 wt. %) were mixed in this weight ratio. Juglone was readily dissolved in the polymer solutions and the common AF biocidal pigment cuprous oxide (Cu_2_O) (powder diameter < 5 µm, Sigma-Aldrich CAS: 1317-39-1) was dispersed in the polymer solutions at ambient temperature by placing the mixtures in glass laboratory bottles (Duran) and rotating these on a laboratory roller mixer (Stuart SRT6D, Cole-Parmer, Staffordshire, UK).

For the coating application, a spin-coating machine (WS-650-23NPP Spin Coater, Laurell Technologies Corporation, North Wales, PA, USA) was used to achieve uniform layers with predictable and reproducible thicknesses. Specifically, the settings for the spin coating were as follows: acceleration: 0–2000 rpm in 10 s, speed: 7500 rpm, time: 1 min, paint viscosity: 500 mPa. To maintain the viscosity, toluene solvent was added as required. Following the spin-coating procedure, the coupons were placed in sterile Petri dishes where they were transferred under sterile conditions (laminar flow hood) to allow solvent evaporation (drying) for 4 days. Following drying, the coupons were carefully partially immersed in 70% EtOH so that the coated surface stayed exposed to air (i.e., only the sides and bottom of the coupons were immersed into the EtOH solution to sterilise them).

### 3.2. Leaching of Juglone from Coatings

To establish calibration curves for juglone, the compound was dissolved in dimethyl sulfoxide (0.5 vol. % of DMSO, all percentages by volume, D8418 Sigma-Aldrich), and serial dilutions of 64 ppm, 32 ppm, 16 ppm, 8 ppm, 4 ppm, 2 ppm, 1 ppm, and 0.5 ppm were prepared in sterile ASW (Sea Salts, S9883, Sigma-Aldrich) [32]. The serial dilutions were then inoculated in a sterile 96-well plate (*n* = 8 per dilution, optical bottom plated with polymer base, clear, Nunc™) and the UV-Vis absorbance spectrum function of a microplate reader (FLUOstar Omega, BMG LABTECH, Offenburg, Germany) was utilised over a scan wavelength range of 200–700 nm at a 2 nm resolution.

In order to assess the leaching of juglone from the current experimental paint matrix, a pilot assay was conducted over 48 h for the coatings 1, 2, 5, and 6 listed in Table 3. Specifically, the coupons were transferred into a clear 24-well plate (Nunclon™ D Multidishes, Sigma-Aldrich) and 1 mL of sterile ASW was added in each well. To avoid water evaporation over the experimental time (48 h), Parafilm^TM^ was used to seal the lid of the 24-well plate. Parafilm^TM^ is a material that is known to allow gas exchange, but not water evaporation. The plate was then incubated in the dark at 28 °C on a rotary shaker at 100 rpm agitation. Each coating formulation was replicated 12 times. The leaching of materials was assessed at two experimental time points, 24 h and 48 h. At each time point, the 24-well plate was removed from the incubator and an aliquot of 200 μm taken from each coupon-containing well was placed in 96-well plates (*n* = 12 per coupon) and UV-Vis optical density measurements were then made at λ = 520 nm using the microplate reader.

### 3.3. Effect of Juglone in Solution against Bacterial Growth (MIC, EC_50_, and MBC)

To establish the effect of juglone in solution, experiments to measure the minimum inhibitory concentration (MIC) were conducted against the marine biofilm-forming species *Cobetia marina* (ATCC 25374). *C. marina* was grown in sterile sea salts and peptone media (sea salts peptone, SSP [15]) overnight under agitation (80 rpm) at 28 °C. MICs were conducted in 96-well plates and bacterial growth was monitored by measuring the optical density at 595 nm (OD_595_). Juglone serial dilutions were prepared as described in the previous section, i.e., 64 ppm, 32 ppm, 16 ppm, 8 ppm, 4 ppm, 2 ppm, 1 ppm, and 0.5 ppm in 0.5% DMSO. There were two controls, one with SSP media plus 0.5% DMSO and one with just SSP media. Each dilution and control was replicated five times while uninoculated wells for each concentration served as blanks and were replicated three times. Measurements were taken every 15 min for 24 h at 28 °C. The MIC was determined as the lowest concentration of juglone at which there was no growth [32]. The effective concentration (EC_50_) was determined as the concentration at which 50% of bacterial growth was inhibited by juglone. Following the 24 h experimental period for the MICs, 5 μL from each well (in triplicates) were transferred onto marine agar to establish the minimum bactericidal concentrations (MBC). Each concentration tested for bacterial growth without the inhibitor (juglone) was replicated three times. The marine agars were then incubated for 24 h and 48 h at 28 °C. Where bacterial colonies were not formed, the MBC was established.

### 3.4. Biofilm Formation on Coatings

**Figure 8:**
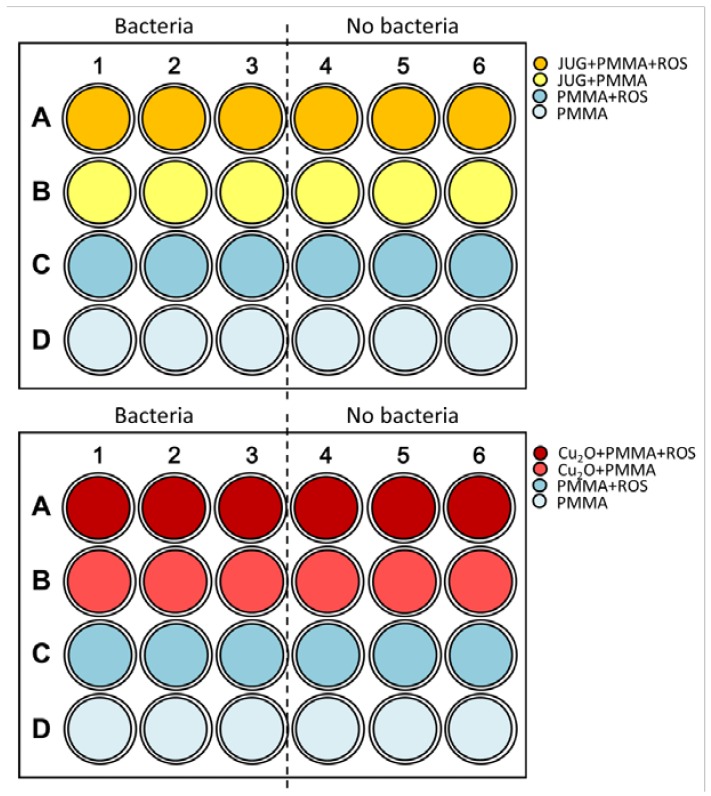
Coupon experimental set-up with coatings (right labels) containing: JUG (**top**) and Cu_2_O (**bottom**). Half of the plates indicate “Bacteria” = wells where bacteria will be inoculated and “No bacteria” wells where no bacteria were added, serving as blanks. JUG = juglone, ROS = rosin, PMMA = poly(methyl methacrylate).

The coated coupons were placed in a 24-well plate (Nunclon™ D Multidishes). The experimental set-up can be seen in Figure 8. The coatings were conditioned for 1 hour with SSP media (950 μL). Following the conditioning time, all coatings were scanned at the LIVE/DEAD wavelengths (i.e., LIVE: λ_EX_ = 485 nm and λ_EM_ = 510 nm, DEAD: λ_EX_ = 540 nm and λ_EM_ = 610 nm, excitation and emission, respectively) to account for any potential autofluorescence deriving from the coatings and correct for any nonbiological signal. After that, the 24-well plate cells containing the coatings were inoculated with 50 μL of *C. marina* grown overnight in SSP (measured OD_595_ = 0.1 at the starting point), as shown in the experimental set up in Figure 8. For each coating formulation, blank coatings were also part of the experimental set up in order to account for any autofluorescence and contaminations (Figure 8). The two 24-well plates were then sealed with Parafilm^TM^ and placed in the dark at 28 °C under agitation (100 rpm) for a total of 48 h.

Following incubation for 48 h, the coupons were removed from the wells and gently rinsed with sterile ASW via pipetting to remove any nonattached cells. To establish planktonic growth at the end-point, OD_595_ measurements were followed. A fluorescent staining method using LIVE/DEAD (FilmTracer™ LIVE/DEAD^®^ Biofilm Viability Kit, Molecular Probes) was performed to assess biofilm growth and cell integrity at the end-point. The LIVE/DEAD kit is comprised of two nucleic acid stains: (1) Syto9, a green fluorescent intercalating membrane permeable stain which is expected to stain all cells and (2) propidium iodide (PI), which is membrane impermeable and is known to stain cells with compromised membranes. The LIVE/DEAD kit was freshly prepared according to the manufacturer’s instructions, i.e., 2 μL of each stain component per 1 mL of phosphate buffer saline, pH 7.4. An aliquot of 500 μL of the LIVE/DEAD mastermix was added to each well ensuring full immersion of the coating in the LIVE/DEAD mixture. The plate was then incubated for 20 min in the dark at 28 °C. At the end of the incubation period, the coupons were gently rinsed twice with ASW to remove excess stain. Finally, 1 mL of ASW was added in order to keep the samples (and subsequently the biofilms) hydrated during the data-acquisition time. The microplate reader (FLUOstar Omega, BMG LABTECH, Offenburg, Germany) was utilised for direct end point measurements (relative fluorescence units, RFU) to allow high-resolution analysis of fluorescence intensity which was conducted using λ_EX_ = 485 nm (for excitation) and λ_EM_ = 510 nm (for emission). Coatings were scanned by the microplate reader and produced a heat map of fluorescence signals from the LIVE/DEAD wavelengths emitted from the coated surfaces using a 30 × 30 well scanning matrix (equivalent to a resolution of 213 × 213 μm, scan width of 6 mm). The software used was Mars Data Analysis Software, Version 2.00 (Omega, BMG LABTECH, Offenburg, Germany).

### 3.5. Microscopy and Image Analysis

CLSM was performed using a Leica TCS SP2 AOBS (Leica Microsystems, Wetzlar, France) microscope. The excitation wavelength used for Syto9 was 480 nm, and the emitted fluorescence was measured in the range of 500–600 nm. The red fluorescent nucleic acid stain PI was excited at 488 nm, and the emitted fluorescence was recorded in the range of 650–700 nm. Images were collected through a Leica water immersion ×63 objective and acquired at 0.502 μm intervals down through the biofilm, and therefore, the number of images in each stack varied according to the thickness of the biofilm. For each coating (1–6, see Table 3), at least two replicas (coupons) were sampled from at least three stacks (i.e., total of *n* > 6). In all experiments, images were acquired from random positions on the coupons. The z-stacks were constructed using the Volocity software (PerkinElmer, Inc., Waltham, MA, USA).

CLSM images were analysed by the computer program COMSTAT [28]. A fixed threshold value and connected volume filtration was used for all image stacks. In-house MATLAB (MathWorks, Natick, MA, USA) scripts were used to obtain data from CLSM images. Features calculated by COMSTAT in the current study:

Biovolume. The biovolume is defined as the average volume of biomass present per unit of substratum area (μm^3^ μm^–2^). It is obtained by multiplying the sum of the biomass pixels in all cross-sectional images of a stack with the voxel size ((pixel size)*_x_* × (pixel size)*_y_* × (pixel size)*_z_*), and dividing the result by the area of the substratum. Apart from the overall volume of the biofilm, the biovolume also provides an estimate of the biomass within the biofilm [28].

Thickness distribution and mean thickness. This function locates the highest point (μm) above each (*x*,*y*) pixel in the bottom layer containing biomass. Hence, thickness is defined as the maximum thickness over a given location, ignoring pores and voids inside the biofilm. The thickness distribution can be used to calculate a range of variables, including biofilm roughness and mean biofilm thickness [28].

### 3.6. Statistical Analysis

Differences between biocide-containing coatings and controls (for both plate reader and CLSM data) were assessed by applying one-way ANOVA. To establish the homogeneity of variances, Levene’s test of equal variances was applied. Where homogeneity of variances was not met, the nonparametric Kruskal–Wallis test was applied. All conclusions are based on 5% level of confidence (*p* < 0.05). Statistical analysis was performed using SPSS version 17.0 and Minitab version 16.

## 4. Conclusions

The use of the fast-tracking plate reader technology combined with nucleic acid staining to assess biofilm formation on coatings has been successful, reproducible, and confirmed by CLSM. CLSM is highly specialised and time consuming for 3D reconstructions (especially if this level of detail is not required), therefore epifluorescence microscopy can be used as a complementary method in future experiments. Model coatings using inert polymer binders were designed containing the NP juglone and the biocide Cu_2_O and compared against controls over 48 h. Coatings containing the NP juglone and the biocide Cu_2_O inhibited biofilm formation after 48 h, when compared to the control coatings that illustrated thick biofilm formation. Juglone has previously shown inhibition of bacterial attachment in short-term experiments [15]. However, this is the first time that this NP was incorporated into a coating system and biofilm formation was assessed. A successful AF technology against biofouling should be able to last for more than five years in order to be economically viable. The main aim is to test whether an AF technology is effective against initial biofilm formation, which takes place within minutes to hours of immersion (for any submerged surface) in the sea, and as such, the methodology developed in the current study illustrated good AF efficacy within the desired time scales. The experimental times could be extended if this is required. AF technologies are now focusing on no-biocide-release coatings due to acknowledged problems with legislative approvals, shifting attention towards foul release coatings and/or nonleaching (“tethered”) biocide coatings [33], as well as topographically modified surfaces [34]. For such technologies, AF activity is aimed to have an immediate response towards fouling. Therefore, testing for initial attachment at short time scales would be appropriate. Thus, the methodology developed in the current study would be ideal for assessing initial biofilm processes, such as bacterial initial attachment and biofilm formation, making this method comparable to realistic scenarios.

## Figures and Tables

**Table 1:** Leaching of juglone from coated surfaces where Coating 1 = JUG + PMMA + ROS and Coating 2 = JUG + PMMA. Each coating was replicated six times (*n* = 6), ±Standard deviation. Significance was assessed using one-way ANOVA. NS = Not Significant; JUG = Juglone, PMMA = poly(methyl methacrylate), ROS = Rosin.

Coating	24 h	48 h	Coating Thickness (μm)	*p*-Value
	Juglone conc. (ppm)			
Coating 1	1.70 ± 0.20	1.96 ± 0.21	14.95 ± 2.02	*p* < 0.05
Coating 2	1.99 ± 0.36	2.35 ± 0.35	19.57 ± 6.05	NS
*p*-value	NS	*p* < 0.04		

**Table 2:** Biovolume and maximum biofilm thickness of *C. marina* found on coated surfaces by COMSTAT analysis [28]. JUG = juglone, PMMA = poly(methyl methacrylate), ROS = rosin.

Formulations	Coating No.	Coating Formulation	Biofilm Max. Thickness (μm)	Biofilm Biovolume (μm^3^ μm^–2^)
NP coating	1	JUG + PMMA + ROS	12.30 ± 0.60	0.09 ± 0.04
	2	JUG + PMMA	12.50 ± 2.30	0.04 ± 0.02
Controls	3	PMMA + ROS	33.50 ± 5.20	8.60 ± 2.17
	4	PMMA	23.00 ± 2.00	1.10 ± 0.49
Biocide coating	5	Cu_2_O + PMMA + ROS	10.90 ± 0.60	0.01 ± 0.00
	6	Cu_2_O + PMMA	09.80 ± 0.70	0.01 ± 0.00
*P_KW_* value	1 vs. 3 * vs. 5		*p <* 0.001	*p <* 0.001
*P_KW_* value	2 vs. 4 * vs. 6		*p <* 0.001	*p* < 0.002
*P_KW_* value	1 vs. 2		NS	NS
*P_KW_* value	3 vs. 4		*p* < 0.043	*p* < 0.005
*P_KW_* value	5 vs. 6		NS	NS

± SE, _KW_ = Kruskal–Wallis, starred coatings (*) signify those that contribute to the effect from post hoc analysis.

**Table 3:** Paint formulations used on glass coupons.

Formulation	Coating No.	Coating Formulation	Coating Composition						Active Compound Concentration (μg/g)	Coating Thickness (μm)	Total No. of Samples
			(wt. %)			(vol. %)					
			AC *	PMMA	ROS	AC *	PMMA	ROS			
NP coatings	1	JUG + PMMA +ROS	6.25	75	18.75	5	76	19	62,500	15.03 ± 4.2	12
	2	JUG + PMMA	6.15	93.85		5	95		61,500	14.78 ± 4.2	12
Biocide coatings	3	Cu_2_O + PMMA + ROS	68.91	24.87	6.22	30	56	14	689,100	7.87 ± 3.0	12
	4	Cu_2_O + PMMA	68.55	34.45		30	70		685,500	4.45 ± 0.7	12
Controls	5	PMMA + ROS	0	80	20	0	80	20	0	3.91 ± 0.7	24
	6	PMMA	0	100		0	100		0	3.81 ± 0.6	24

JUG = juglone, ROS = rosin, PMMA = poly(methyl methacrylate), NP= Natural Product (Juglone), Blanks: uninoculated, i.e., there were no bacteria present. * AC = Active Compound (either Juglone or Cu_2_O).
